# Different effect of hypo- and hypermetabolism on cognition in dementia with Lewy bodies: are they coupled or independent?

**DOI:** 10.1038/s41531-023-00622-w

**Published:** 2024-01-03

**Authors:** Seong Ho Jeong, Jungho Cha, Han Soo Yoo, Seok Jong Chung, Jin Ho Jung, Young H. Sohn, Phil Hyu Lee

**Affiliations:** 1 Department of Neurology, Inje University Sanggye Paik Hospital, Seoul, South Korea; 2 Department of Neurology, Yonsei University College of Medicine, Seoul, South Korea; 3 Nash Family Center for Advanced Circuit Therapeutics, Icahn School of Medicine at Mount Sinai, New York, NY USA; 4 Department of Neurology, Gangnam Severance Hospital, Yonsei University College of Medicine, Seoul, South Korea; 5 Department of Neurology, Yongin Severance Hospital, Yonsei University Health System, Yongin, South Korea; 6 Department of Neurology, Inje University Busan Paik Hospital, Busan, South Korea; 7 Severance Biomedical Science Institute, Yonsei University College of Medicine, Seoul, South Korea

**Keywords:** Prognostic markers, Cognitive ageing, Neurodegeneration

## Abstract

Patients with dementia with Lewy bodies (DLB) show widespread brain metabolic changes. This study investigated whether brain hypo- and hypermetabolism in DLB have differential effects on cognition. We enrolled 55 patients with DLB (15 prodromal DLB [MCI-LB] and 40 probable DLB) and 13 healthy controls who underwent ^18^F-fluorodeoxyglucose positron emission tomography and detailed neuropsychological tests. Metabolic indices reflecting associated changes in regional cerebral glucose metabolism were calculated as follows: index^(-)^ for hypometabolism [DLB-hypo] and index^(+)^ for hypermetabolism [DLB-hyper]. The effects of DLB-hypo or DLB-hyper on cognitive function were assessed using a multivariate linear regression model. Additionally, a linear mixed model was used to investigate the association between each index and the longitudinal cognitive decline. There was no correlation between DLB-hypo and DLB-hyper in the disease group. The multivariate linear regression model showed that DLB-hypo was associated with language, visuospatial, visual memory, and frontal/executive functions; whereas DLB-hyper was responsible for attention and verbal memory. There was significant interaction between DLB-hypo and DLB-hyper for verbal and visual memory, which was substantially affected by DLB-hyper in relatively preserved DLB-hypo status. A linear mixed model showed that DLB-hypo was associated with longitudinal cognitive outcomes, regardless of cognitive status, and DLB-hyper contributed to cognitive decline only in the MCI-LB group. The present study suggests that DLB-hypo and DLB-hyper may be independent of each other and differentially affect the baseline and longitudinal cognitive function in patients with DLB.

## Introduction

Dementia with Lewy bodies (DLB) is the second most common neurodegenerative dementia, which is characterized by cognitive decline with several core features including fluctuation of attention, vivid visual hallucination (VH), rapid eye movement sleep behavior disorder (RBD), and parkinsonism^1^. The newly updated 2017 diagnostic criteria explicitly specified clinical features and diagnostic biomarkers to provide diagnostic guidance; however, it is still challenging to diagnose DLB in the real world^1,2^.

Glucose metabolic patterns assessed by ^18^F-fluorodeoxyglucose (FDG) positron emission tomography (PET) exhibiting relative hypometabolism in the temporo-parieto-occipital area and relative hypermetabolism in the medial temporal lobe, orbitofrontal cortex, pontocerebellum, striatum, and sensorimotor cortex. Metabolic brain imaging is a promising diagnostic biomarker for DLB^3^. Previous studies showed that metabolic changes are associated with core features and cognition in patients with DLB^4^. However, although the areas showing relative hypometabolism and hypermetabolism have been detected concurrently in individual patients with DLB^5,6^, no study has investigated whether the hypo- and hyper-metabolic areas are coupled or independent of each other. In addition, the different roles of hypometabolism and hypermetabolism in cognition have not yet been investigated in DLB. Recently, a study showed that hypermetabolism in the cerebellum is associated with cognition independent of hypometabolism in patients with Parkinson’s disease (PD)^7^, which shares similar neuropathological findings with DLB.

In the present study, we hypothesized that relative brain hypometabolism and hypermetabolism are independently associated with cognition in patients with DLB. Therefore, we investigated the relationship between glucose metabolism in the hypo- and hyper-metabolic regions, baseline cognitive function, and longitudinal cognitive performance in patients with DLB spectrum encompassing normal aging, prodromal DLB (MCI-LB), and probable DLB.

## Results

### Demographic and clinical characteristics of the participants

The demographic and clinical characteristics of the normal control (*n* = 13), MCI-LB (*n* = 15), and DLB (*n* = 40) groups are summarized in Table 1. Age and years of education did not differ among the groups, whereas the proportion of female patients in the MCI-LB group was lower than that in the other groups. There were no significant differences among the three groups in terms of vascular risk factors. The proportion of clinical core features was comparable between the MCI-LB and DLB groups. As expected, MMSE scores, CDR-SOB scores, and standardized z-scores for all items were lowest in the DLB group. In terms of glucose metabolism, the MCI-LB and DLB groups had significantly lower DLB-hypo and DLB-hyper than the control group, whereas the DLB-hypo or DLB-hyper groups did not differ between the MCI-LB and DLB groups.

**Table 1:** Demographic and clinical characteristics of study subjects.

Control		MCI-LB	DLB	*P* value
**Number**	13	15	40	
**Demographics**				
Age, year	71.85 ± 4.20	75.19 ± 6.34	75.58 ± 7.56	0.228
Sex, female (%)	9 (69.2)	3 (20.0)	23 (57.5)	**0.017**^a,b^
Education, year	14.00 ± 3.72	11.73 ± 4.18	10.41 ± 5.62	0.086
**LBD features**				
Cognitive fluctuation	NA	9 (60.0)	21 (52.5)	0.847
Visual hallucination	NA	6 (40.0)	18 (45.0)	0.978
Parkinsonism	NA	10 (66.7%)	28 (70.0%)	>0.999
UPDRS motor score	NA	17.47 ± 5.84	22.78 ± 12.31	0.116
RBD	NA	10 (66.7)	23 (57.5)	0.757
**Vascular risk factors,** ***n*** **(%)**				
Hypertension	5 (38.5)	6 (40.0)	20 (50.0)	0.704
Diabetes mellitus	3 (23.1)	3 (20.0)	10 (25.0)	0.926
Dyslipidemia	6 (46.2)	5 (33.3)	12 (30.0)	0.564
**Neuropsychological tests**				
Item				
Digit span forward	0.43 ± 0.95	−0.03 ± 0.76	−0.38 ± 1.04	**0.032**^c^
Digit span backward	0.95 ± 1.59	−0.33 ± 0.88	−1.05 ± 1.32	**<0.001**^a,c^
K-BNT	0.35 ± 0.73	−0.47 ± 1.01	−2.00 ± 1.98	**<0.001**^b,c^
RCFT copy	0.33 ± 0.64	−0.84 ± 1.58	−3.66 ± 4.51	**0.001**^b,c^
SVLT immediate recall	0.97 ± 0.88	−1.23 ± 0.88	−1.56 ± 0.96	**<0.001**^a,c^
SVLT delayed recall	0.93 ± 0.92	−1.41 ± 0.90	−1.96 ± 0.76	**<0.001**^a,c^
SVLT recognition	0.77 ± 0.74	−1.52 ± 1.31	−2.06 ± 1.54	**<0.001**^a,c^
RCFT immediate recall	0.04 ± 0.81	−1.12 ± 0.78	−1.61 ± 0.64	**<0.001**^a,c^
RCFT delayed recall	0.19 ± 0.65	−1.19 ± 0.85	−1.77 ± 0.73	**<0.001**^a,b,c^
RCFT recognition	−0.30 ± 0.85	−0.78 - ± 1.32	−1.82 ± 1.26	**<0.001**^b,c^
COWAT animal	0.51 ± 1.27	−0.99 ± 1.09	−1.60 ± 0.84	**<0.001**^a,c^
COWAT supermarket	−0.02 ± 0.81	−1.14 ± 0.54	−1.52 ± 0.86	**<0.001**^a,c^
COWAT phonemic	0.28 ± 0.81	−1.05 ± 0.98	−1.54 ± 0.83	**<0.001**^a,c^
Stroop color reading	0.09 ± 0.97	−1.51 ± 1.47	−2.62 ± 1.19	**<0.001**^a,b,c^
K-MMSE score	28.92 ± 1.12	25.60 ± 2.06	19.48 ± 4.73	**<0.001**^a,b,c^
CDR-SOB	0.19 ± 0.25	1.77 ± 0.84	5.16 ± 2.77	**<0.001**^a,b,c^
**FDG-PET**				
DLB-hypo	1.28 ± 0.04	1.17 ± 0.07	1.14 ± 0.08	**<0.001**^a,c^
DLB-hyper	0.88 ± 0.04	0.96 ± 0.03	0.98 ± 0.05	**<0.001**^a,c^

Values are expressed as mean ± standard deviation or number (percentage). *P*-values are the results of analyses of variance, chi-square tests, or Fisher’s exact tests, as appropriate.

*CDR-SOB* Clinical Dementia Rating-Sum of Boxes, *COWAT* Controlled Oral Word Association Test, *DLB* dementia with Lewy bodies, *DLB-hypo* hypometabolic changes in dementia with Lewy bodies, *DLB-hyper* hypermetabolic changes in dementia with Lewy bodies, *FDG* ^18^F-fluorodeoxyglucose, *K-BNT* Korean version of the Boston Naming Test, *K-MMSE* Korean version of Mini-Mental State Examination, *MCI-LB* prodromal dementia with Lewy bodies, *RBD* rapid eye movement sleep behavior disorder, *SVLT* Seoul Verbal Learning Test, *UPDRS* Unified Parkinson’s Disease Rating Scale, *RCFT* Rey-Osterrieth Complex Figure Test

^a^Significantly different in comparison between control and MCI-LB groups.

^b^Significantly different in comparison between MCI-LB and DLB groups.

^c^Significantly different in comparison between control and DLB groups.

Bold values identify statistical significance (*p* < 0.05).

### Characteristics of the DLB-hypo and DLB-hyper

**Fig. 1:**
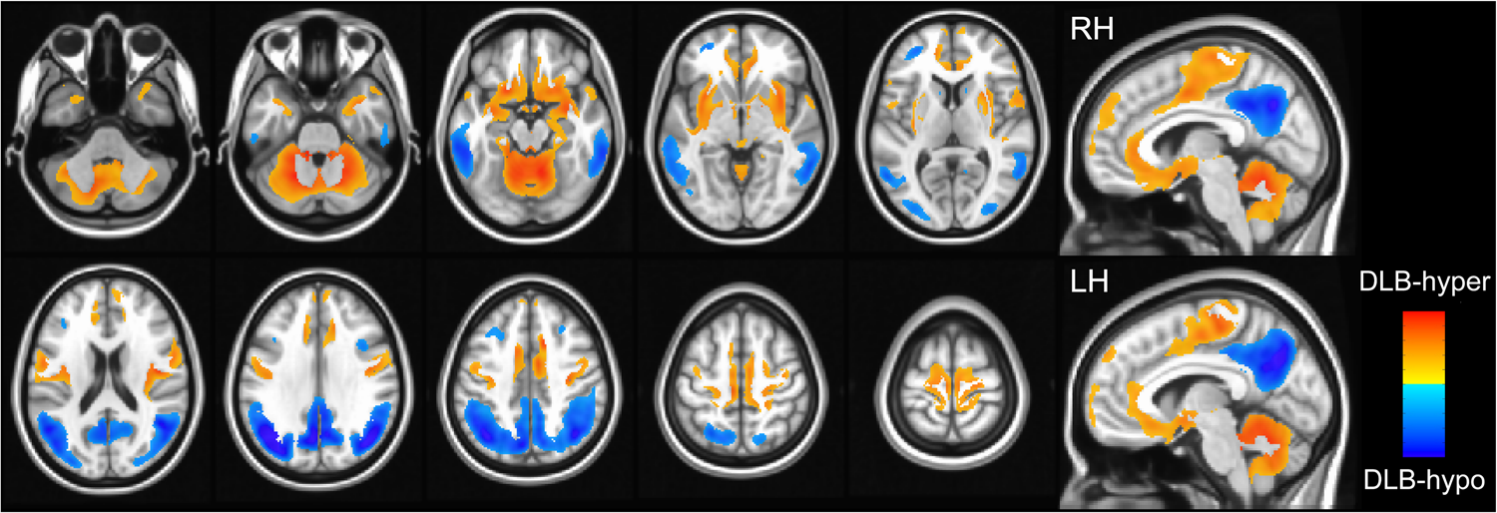
Topography of DLB-hypo and DLB-hyper. DLB-hypo was characterized by relatively reduced bilateral metabolic activity (color-coded blue) in the bilateral parietal, temporal, and occipital cortices, while DLB-hyper showed relatively increased metabolic activity (color-coded red) in the cerebellum, bilateral sensorimotor, orbitofrontal, insular, and parahippocampal cortices, bilateral putamen, globus pallidum, hippocampus, and amygdala. DLB-hypo hypometabolic changes in dementia with Lewy bodies, DLB-hyper hypermetabolic changes in dementia with Lewy bodies.

The clusters of relative hypometabolism (DLB-hypo) and hypermetabolism (DLB-hyper) in the comparison between the normal control and DLB group are illustrated in Fig. 1. Using global mean normalization, clusters of significant relative hypometabolism in the gray matter were detected in the bilateral parietal, temporal, and occipital cortices of DLB patients compared with normal controls. Significant gray matter relative hypermetabolism was observed in the cerebellum including vermis and bilateral cerebellar cortices, bilateral sensorimotor, orbitofrontal, insular, and parahippocampal cortices, bilateral putamen, globus pallidus, hippocampus, and amygdala. Correlation analysis showed a negative relationship between DLB-hypo and DLB-hyper in all participants (i.e. healthy controls, MCI-LB, and DLB groups; *r* = −0.440, *P* < 0.001), whereas there was no association between them within the whole DLB group (i.e. MCI-LB and DLB groups; *r* = −0.219, *P* = 0.108).

### Association between DLB-hypo or DLB-hyper and cognitive function

Multivariate linear regression analyses for MMSE scores showed that DLB-hypo (*β* = 0.46, *P* = 0.001), not DLB-hyper (*β* = −0.21, *P* = 0.108), was significantly associated with MMSE scores in all participants after adjusting for age at FDG scan, sex, years of education, and ICV. This result maintained in the whole DLB groups (DLB-hypo, *β* = 0.45, *P* = 0.017; DLB-hyper, *β* = −0.03, *P* = 0.836; Table 2). The results of the linear regression analyses performed to determine the impact of DLB-hypo or DLB-hyper on each item of neuropsychological tests are summarized in Table 3. Multivariate linear regression analyses using the backward elimination method showed that only DLB-hypo was selected or significant in the models for digit span forward (*β* = 0.36, *P* = 0.003) and backward (*β* = 0.28, *P* = 0.028), K-BNT (*β* = 0.41, *P* = 0.001), RCFT copy (*β* = 0.56, *P* < 0.001), COWAT supermarket (*β* = 0.30, *P* = 0.015), and COWAT phonemic (*β* = 0.46, *P* = 0.002) tests. In contrast, only the DLB-hyper was selected for RCFT recognition (*β* = −0.34, *P* = 0.005). Although both DLB-hypo and DLB-hyper were selected and significant in the models for immediate and delayed recall, recognition of SVLT, and immediate and delayed recall of RCFT, DLB-hypo showed the greater absolute value of standardized beta coefficients in the models of RCFT immediate (*β* = 0.42, *P* < 0.001) and delayed recall (*β* = 0.51, *P* < 0.001) than those of DLB-hyper (*β* = −0.30, *P* = 0.007; *β* = −0.27, *P* = 0.039), while DLB-hyper had the greater absolute *β* values in the models of SVLT immediate recall (*β* = −0.53, *P* < 0.001), delayed recall (*β* = −0.44, *P* < 0.001), and recognition (*β* = −0.48, *P* < 0.001) than those of DLB-hypo (*β* = 0.29, *P* = 0.004; *β* = 0.35, *P* = 0.001; *β* = 0.25, *P* = 0.022). We determined that the variance inflation factor for each variable was < 10, indicating that multicollinearity did not occur.

**Table 2:** Multivariate linear regression analyses for the effect of DLB-hypo and/or DLB-hyper on MMSE scores.

	Selected variables	*β*	*P value*	*VIF*	*F (P)*	*Adj. R*^*2*^
All participants (HC, MCI-LB, and DLB groups)	Age at FDG scan	−0.16	0.213	1.80	8.86 ( < 0.001)	0.41
	Sex	−0.18	0.096	1.33		
	Years of education	0.38	<0.001	1.29		
	ICV	−0.01	0.955	1.12		
	**DLB-hypo**	**0.46**	**0.001**	2.04		
	DLB-hyper	−0.21	0.108	1.89		
Whole DLB groups (MCI-LB and DLB groups)	Age at FDG scan	−0.20	0.242	2.02	3.60 (0.005)	0.22
	Sex	−0.34	0.024	1.52		
	Years of education	0.30	0.076	1.91		
	ICV	0.009	0.945	1.18		
	**DLB-hypo**	**0.451**	**0.017**	2.32		
	DLB-hyper	−0.030	0.836	1.43		

The results of multivariate linear regression models after adjusting for age at FDG scan, sex, years of education, ICV, DLB-hypo, and DLB-hyper as independent variables.

*β* standardized beta coefficient, *DLB* dementia with Lewy bodies, *DLB-hypo* hypometabolic changes in dementia with Lewy bodies, *DLB-hyper* hypermetabolic changes in dementia with Lewy bodies, *HC* healthy control, *ICV* intracranial volume, *MCI-LB* prodromal dementia with Lewy bodies.

Bold values identify statistical significance (*p* < 0.05).

**Table 3:** Multivariate linear regression analyses for the effect of DLB-hypo and/or DLB-hyper on each item in neuropsychological test.

Cognitive item	Selected variables	*β*	*P value*	*F (P)*	*Adj. R*^*2*^
Digit span forward	Years of education	0.27	0.023	6.40 (0.003)	0.14
**DLB-hypo**		0.36	**0.003**		
Digit span backward	Years of education	0.20	0.084	5.74 (0.002)	0.18
**DLB-hypo**		0.28	**0.028**		
DLB-hyper		−0.24	0.057		
K-BNT	Sex	−0.33	0.006	7.32 (<0.001)	0.27
Years of education		−0.19	0.113		
**DLB-hypo**		0.41	**0.001**		
DLB-hyper		−0.21	0.084		
RCFT copy	Sex	−0.22	0.049	11.37 (<0.001)	0.38
Years of education		−0.15	0.170		
**DLB-hypo**		0.56	**<0.001**		
DLB-hyper		−0.16	0.154		
SVLT immediate recall	**DLB-hypo**	0.29	**0.004**	32.16 (<0.001)	0.48
**DLB-hyper**		−0.53	**<0.001**		
SVLT delayed recall	Years of education	0.16	0.093	17.95 (<0.001)	0.43
**DLB-hypo**		0.35	**0.001**		
**DLB-hyper**		−0.44	**<0.001**		
SVLT recognition	**DLB-hypo**	0.25	**0.022**	21.47 (<0.001)	0.38
	**DLB-hyper**	−0.48	**<0.001**		
RCFT immediate recall	**DLB-hypo**	0.42	**<0.001**	19.48 (<0.001)	0.36
**DLB-hyper**		−0.30	**0.007**		
RCFT delayed recall	Age at FDG scan	−0.18	0.154	16.2 (<0.001)	0.41
	**DLB-hypo**	0.51	**<0.001**		
	**DLB-hyper**	−0.27	**0.039**		
RCFT recognition	**DLB-hyper**	−0.34	**0.005**	8.53 (0.005)	0.10
COWAT animal	Sex	−0.17	0.143	9.89 (<0.001)	0.35
Years of education		0.22	0.047		
**DLB-hypo**		0.35	**0.003**		
**DLB-hyper**		−0.35	**0.002**		
COWAT supermarket	Sex	0.19	0.092	7.58 (<0.001)	0.23
**DLB-hypo**		0.30	**0.015**		
**DLB-hyper**		−0.20	0.100		
COWAT phonemic	Age at FDG scan	−0.19	0.158	11.42 (<0.001)	0.32
**DLB-hypo**		0.46	**0.002**		
**DLB-hyper**		−0.23	0.093		
Stroop color reading	Sex	0.16	0.126	13.64 (<0.001)	0.36
**DLB-hypo**		0.25	**0.029**		
**DLB-hyper**		−0.42	**<0.001**		

The results of multivariate linear regression models using backward elimination method including age at FDG scan, sex, years of education, intracranial volume, DLB-hypo, and DLB-hyper as independent variables.

*β* standardized beta coefficient, *COWAT* Controlled Oral Word Association Test, *DLB-hypo* hypometabolic changes in dementia with Lewy bodies, *DLB-hyper* hypermetabolic changes in dementia with Lewy bodies, *K-BNT* Korean version of the Boston Naming Test, *RCFT* Rey-Osterrieth Complex Figure Test, *SVLT* Seoul Verbal Learning Test.

Bold values identify statistical significance (*p* < 0.05).

### Interaction effect between DLB-hypo or DLB-hyper on cognitive function

The equation for the interaction analysis was given by Eq. (1).

1 $$\begin{array}{l}{Composite\; score}\,{of}\,{each}\,{cognitive}\,{domain}=\beta 0+\beta 1\times {age}+\beta 2\times {sex}\\+\,\beta 3\times {years}\,{of}\,{education}+\beta 4\times {ICV}+\beta 5\times {DLB}-{hypo}\\+\,\beta 6\times {DLB}-{hyper}+\beta 7\times ({DLB}-{hypo}\times {DLB}-{hyper})\end{array}$$

**Fig. 2:**
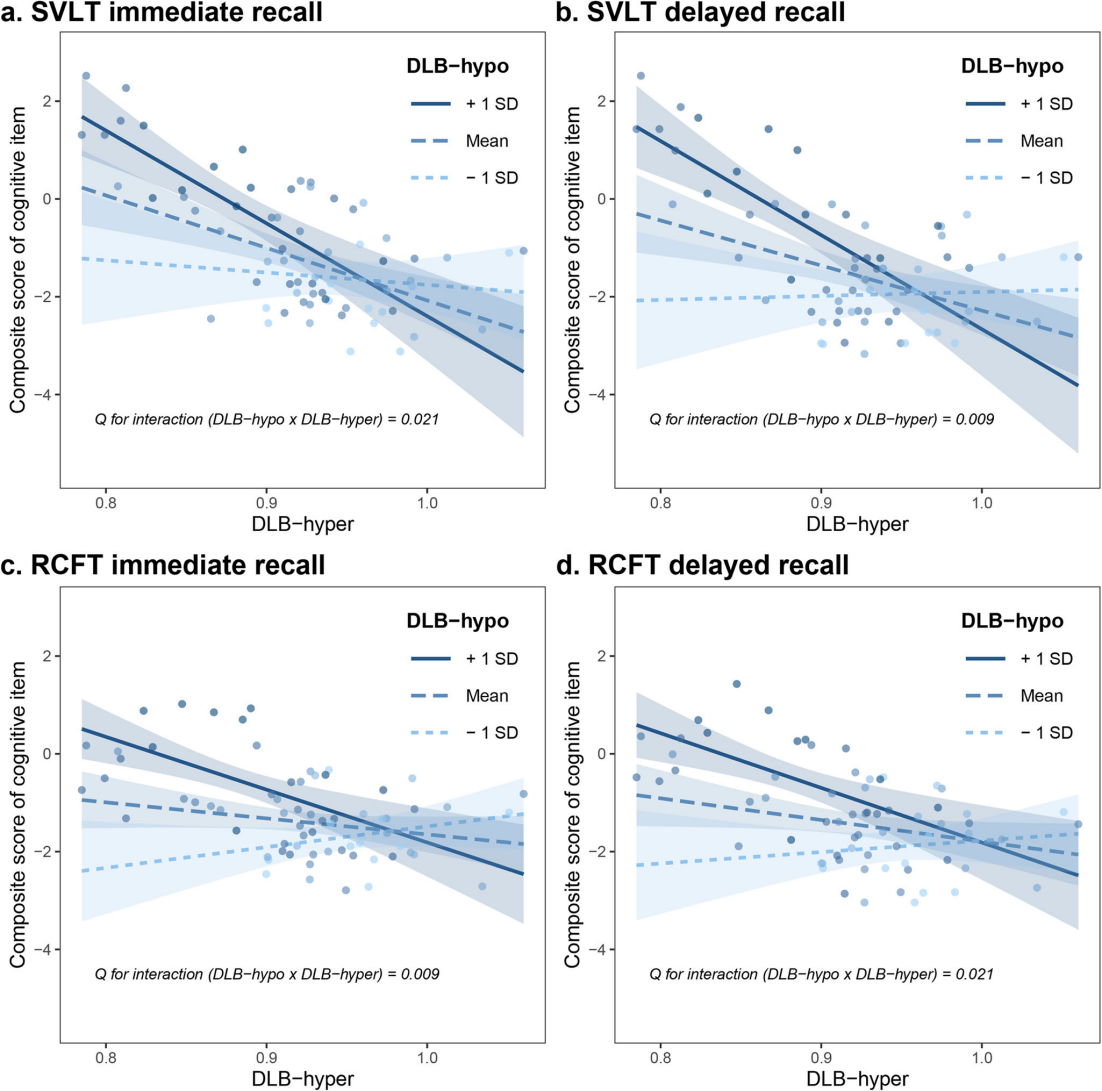
Interaction plot between DLB-hypo and DLB-hyper on each cognitive item. Three lines each indicate the regression line between the composite score of each cognitive item (**a** SVLT immediate recall, **b** SVLT delayed recall, **c** RCFT immediate recall, and **d** RCFT delayed recall) and DLB-hyper under 1 SD above or below the mean and the mean itself of DLB-hypo. DLB-hypo hypometabolic changes in dementia with Lewy bodies, DLB-hyper hypermetabolic changes in dementia with Lewy bodies, RCFT Rey-Osterrieth Complex Figure Test, SD standard deviation, SVLT Seoul Verbal Learning Test.

The results of the interaction analysis of cognitive function are presented in Supplementary Table 1. The interaction term between DLB-hypo and DLB-hyper was significant for SVLT immediate (*β* = −5.633, *Q* = 0.021) and delayed recall (*β* = −6.691, *Q* = 0.009) and RCFT immediate (*β* = −7.333, *Q* = 0.009) and delayed recall (*β* = −5.918, *Q* = 0.021). Next, we illustrate the results of the interaction term using an interaction plot according to the status of DLB-hypo. In the status of relatively preserved DLB-hypo (i.e., mild hypometabolism), which was represented by 1 SD above the mean, each cognitive item of SVLT and RCFT recall was substantially affected by the severity of DLB-hyper. In contrast, each cognitive item was minimally affected by the severity of DLB-hyper when DLB-hypo was severely decreased (represented by 1 SD below the mean) (Fig. 2).

### Longitudinal assessment of the changes in MMSE scores

The subsample of 48 participants [MCI-LB (*n* = 15) and DLB (*n* = 33)] who had at least two MMSE scores with a 1-year interval had demographic and clinical characteristics similar to those of the participants in this study (Supplementary Table 2). The equation of linear mixed model was given by Eqs. (2), (3), and (4).

2 $$\begin{array}{l}{Model}\,1:{MMSE}\,{score} \sim \beta 0+(\beta 1\times {age})+(\beta 2\times {sex})+(\beta 3\times {years}\,{of}\,{education})\\+\,(\beta 4\times {cognitive}\,{status}[{dementia}\,{vs}.{non}-{dementia}])+\,(\beta 5\times {ICV})\\+\,(\beta 6\times {DLB}-{hypo})+(\beta 7\times {time})+\,(\beta 8\times {DLB}-{hypo}\times {time})\end{array}$$

3 $$\begin{array}{ll}{Model}\,2:{MMSE}\,{score} \sim \beta 0+(\beta 1\times {age})+(\beta 2\times {sex})\\+\,(\beta 3\times {years\; of}\,{education})+(\beta 4\times {cognitive}\,{status}[{dementia}\,{vs}.{non}-{dementia}])\\+\,(\beta 5\times {ICV})+(\beta 6\times {DLB}-{hyper})+(\beta 7\times {time})+(\beta 8\times {DLB}-{hyper}\times {time})\end{array}$$

4 $$\begin{array}{ll}{Model}\,3:{MMS\; score} \sim \beta 0+(\beta 1\times {age})+(\beta 2\times {sex})+(\beta 3\times {years}\,{of}\,{education})\\+\,(\beta 4\times {cognitive}\,{status}[{dementia}\,{vs}.{non}-{dementia}])+(\beta 5\times {ICV})\\+\,(\beta 6\times {DLB}-{hypo})+(\beta 7\times {DLB}-{hyper})+(\beta 8\times {time})\\+\,(\beta 9\times {DLB}-{hypo}\times {time})+(\beta 10\times {DLB}-{hyper}\times {time})\end{array}$$

There was a significant positive value for the DLB-hypo × time interaction term in the linear mixed model of MMSE total score (estimates = 1.16, SE = 0.28, *P* < 0.001, Table 4, *Model 1*), indicating that the longitudinal MMSE score decrement per year was approximately 1.16 smaller per 1 SD increase in baseline DLB-hypo. In terms of DLB-hyper, the DLB-hyper × time interaction term was also significant (estimates = −0.85 SE = 0.23, *P* < 0.001, Table 4, *Model 2*), indicating that the MMSE score decline per year was approximately 0.85 greater per 1 SD increase in baseline DLB-hyper. In the two-way interaction model (*Model 3*), both the DLB-hypo × time (estimates = 0.89, SE = 0.29, *P* = 0.003) and DLB-hyper × time (estimates = −0.65, SE = 0.24, *P* = 0.007) interaction terms were significant, indicating that the effects of DLB-hypo or DLB-hyper on longitudinal MMSE decrement may be independent of each other (Table 4). In the subgroup analyses according to cognitive status, DLB-hypo × time and DLB-hyper × time interaction terms in the linear mixed model for longitudinal changes in MMSE scores were significant in the MCI-LB group (*Model 1*, estimates = 1.71, SE = 0.64, *P* = 0.010; *Model 2*, estimates = −1.27, SE = 0.48, *P* = 0.011), both of which were still significant in *Model 3* (DLB-hypo × time, estimates = 1.50, SE = 0.61, *P* = 0.017; DLB-hyper × time, estimates = −1.12, SE = 0.46, *P* = 0.020). In contrast, although the DLB-hypo × time and DLB-hyper × time interaction terms were significant in the DLB group (*Model 1*, estimates = 1.07, SE = 0.31, *P* = 0.001; *Model 2*, estimates = −0.70, SE = 0.28, *P* = 0.014), only the DLB-hypo × time interaction term was significant in *Model 3* (DLB-hypo × time, estimates = 0.85, SE = 0.34, *P* = 0.014; DLB-hyper × time, estimates = −0.39, SE = 0.29, *P* = 0.182, Supplementary Table 3).

**Table 4:** Longitudinal models predicting change in K-MMSE score over time.

	*Model 1*^a^		*Model 2*^b^		*Model 3*^c^	
	Estimates (SE)	*P*	Estimates (SE)	*P*	Estimates (SE)	*P*
Intercept	47.93 (12.05)	<0.001	35.64 (11.10)	0.003	44.84 (11.33)	<0.001
Age	−0.05 (0.10)	0.599	0.14 (0.08)	0.086	0.01 (0.10)	0.935
Sex, female vs. male	−2.06 (1.43)	0.158	−1.89 (1.38)	0.180	−1.83 (1.34)	0.180
Education	0.18 (0.14)	0.223	0.18 (0.14)	0.186	0.27 (0.14)	0.062
Cognitive status, dementia vs. non-dementia	−6.15 (1.37)	<0.001	−5.76 (1.30)	<0.001	−5.64 (1.29)	<0.001
Intracranial volume	−4.39 (5.46)	0.427	−6.34 (5.39)	0.247	−6.67 (5.24)	0.210
DLB-hypo, per 1 SD increase	1.03 (0.98)	0.299			0.83 (0.96)	0.393
DLB-hyper, per 1 SD increase			−1.17 (0.90)	0.199	−0.88 (0.90)	0.337
Time, y	−1.26 (0.19)	<0.001	−1.19 (0.20)	<0.001	−1.16 (0.19)	<0.001
DLB-hypo × time	**1.16 (0.28)**	**<0.001**			**0.89 (0.29)**	**0.003**
DLB-hyper × time			**−0.85 (0.23)**	**<0.001**	−**0.65 (0.24)**	**0.007**

Data are the results of the linear mixed model for the change in K-MMSE score over time.

*DLB-hypo* hypometabolic changes in dementia with Lewy bodies, *DLB-hyper* hypermetabolic changes in dementia with Lewy bodies, *K-MMSE* the Korean version of the mini-mental status examination, *SE* standard error.

^a^Model 1 is the result of the linear mixed model after adjusting for age, sex, education, cognitive status, intracranial volume, DLB-hypo, time, and DLB-hypo × time.

^b^Model 2 is the result of the linear mixed model after adjusting for age, sex, education, cognitive status, intracranial volume, DLB-hyper, time, and DLB-hyper × time.

^c^Model 3 is the result of the linear mixed model after adjusting for age, sex, education, cognitive status, intracranial volume, DLB-hypo, DLB-hyper, time, DLB-hypo × time, and DLB-hyper × time.

Bold values identify statistical significance (*p* < 0.05).

## Discussion

In the present study, we separately analyzed the relative brain hypometabolism and hypermetabolism in DLBRP and investigated their different effects on baseline and longitudinal cognitive function in patients with DLB. Our major findings were as follows: First, DLB-hypo contributed to dysfunction of language, visuospatial, visual memory, and frontal/executive domains, whereas DLB-hyper was more strongly associated with dysfunction in attention and verbal memory domains. Second, there was a significant interaction effect between DLB-hypo and DLB-hyper for verbal and visual memory, which was substantially affected by DLB-hyper in the relatively preserved DLB-hypo status. Third, both DLB-hypo and DLB-hyper have detrimental effects on longitudinal cognition, and their effects may be independent of each other. Taken together, these findings suggest that DLB-hypo and DLB-hyper may differentially and independently affect baseline and longitudinal cognitive functions in patients with DLB.

We found that both DLB-hypo and DLB-hyper were closely associated with all cognitive items. Specifically, DLB-hypo was more relevant to language, visuospatial, visual memory, and frontal executive domains, whereas DLB-hyper was more closely associated with attention and verbal memory function. Generally, hypometabolism in the neurodegenerative disorder reflects neuronal dysfunction and previous studies showed its usefulness as diagnostic and predictive biomarkers^3,8,9^. Our previous study also showed that hypometabolism is significantly associated with cognitive dysfunction in patients with DLB^10^. Conversely, the role of hypermetabolism in neurodegenerative disorders remains unclear. Hypermetabolism is regarded as a compensatory metabolic changes^11,12^, and the effect of hypermetabolism on cognition has been rarely investigated before. This is the first study that revealed that hypermetabolism in DLB may not be just confined to the result of compensation but may independently affect cognitive dysfunction. Indeed, previous studies reported that dopamine deficiency in dopaminergic inhibitory neurocircuit would result in enhanced oscillatory burst activity of the basal ganglia^13,14^, leading to increased brain metabolism in areas exhibiting PD-related pattern activity, which was ameliorated after levodopa infusion^15^. Next, we further conducted interaction analyses between DLB-hypo and DLB-hyper for each cognitive items. There was a significant interaction between DLB-hypo and DLB-hyper for the verbal and visual memory items. In a schematic illustration of these interaction effects (Fig. 2), each item of the verbal and visual memory was substantially negatively affected by DLB-hyper when DLB-hypo was relatively mild. In contrast, DLB-hyper was not significantly associated with memory function in severely decreased DLB-hypo status. Although no previous studies have investigated the role of brain hypermetabolism in DLB, some studies have suggested that brain hyperactivity is related to cognition and prognosis in other neurodegenerative disorders. In amyotrophic lateral sclerosis and Huntington’s disease, hypermetabolism has been shown to affect motor prognosis in several previous studies^12,16^. A recent study showed that cerebellar hypermetabolism is closely related to cognitive impairment in PD^7^. Another study demonstrated that brain hypermetabolism in the frontal, lateral temporal, and posterior parietal regions is related to tau deposition and episodic memory function in patients with MCI with a low amyloid burden^17^. In this regard, because brain hypometabolism is associated with the clinical consequences of neurodegeneration^8,18,19^, the present study suggests that DLB-hyper may have a detrimental effect on memory function especially in the early stage of DLB.

In the longitudinal analysis, a linear mixed model showed that both DLB-hypo and DLB-hyper were associated with longitudinal cognitive outcomes, and that their effects may be independent of each other. Interestingly, when a subgroup analysis was performed according to cognitive status, DLB-hypo was associated with cognitive decline irrespective of cognitive status, whereas DLB-hyper was associated with longitudinal MMSE score changes only in the MCI-LB subgroup. One previous study on Down syndrome showed that temporal cortex hypermetabolism precedes the onset of dementia and this hypermetabolism diminished thereafter^20^. Another study revealed that glucose metabolism in the hippocampal formation was negatively associated with cognitive function in cognitively impaired patients, which suggested that relative hippocampal hypermetabolism would be the result of detrimental maladaptation^21^. In line with previous studies^20,21^, the result of the present study suggests that hypermetabolism plays a role in compensatory changes and affects longitudinal cognitive prognosis in patients with early-stage DLB. Alternatively, hypermetabolism may be pathogenetically associated with neuronal activity, which is prone to the spread of toxic proteins. For example, tau pathology-associated regional hypermetabolism observed in patients with MCI^17^ may reflect enhanced neuronal activity that induces propagation of toxic tau protein^22^. Regarding that the transmission of α-synuclein, which is the major pathophysiology of Lewy body spectrum disorder, is also affected by neuronal activity^23^, it is speculated that hypermetabolism may reflect neuronal hyperactivity that would play a crucial role in α-synuclein propagation in the early stage of DLB. Accordingly, future studies should investigate whether the therapeutic targets of DLB-hyper can improve cognition and prognosis in the early stages of DLB.

Most previous studies have examined the pattern of cerebral glucose metabolism without considering independent role of hypermetabolism and hypometabolism in cognitive function in Lewy body spectrum disorders (i.e., PD, DLB, and idiopathic RBD)^24–28^. Consistent with previous reports^3,5^, we found hypometabolic areas in the bilateral parietal, temporal, and occipital cortices and hypermetabolic areas in the cerebellum, bilateral sensorimotor, orbitofrontal, insular, and parahippocampal cortices, bilateral putamen, globus pallidus, hippocampus, and amygdala. However, although DLB-hypo and DLB-hyper were negatively associated in all participants including healthy controls, there was no significant correlation between DLB-hypo and DLB-hyper within the whole DLB group. Moreover, our data showed that the association between general cognition and hypo- and hyper-metabolic patterns differed depending on cognitive status, and the two metabolic patterns were differentially and independently associated with baseline and longitudinal cognitive function in patients with DLB. The results of the present study suggest that these two metabolic patterns should be considered separately in future studies of Lewy body spectrum disorders. Due to small number of participants, we did not validate our findings in a cohort, not used to derive the metabolic indices. This and the fact that this was a single centre study limits the generalizability of our findings. However, recent studies have consistently shown that hypo- and hypermetabolism in DLB are not mutually exclusive^4,29^. Considering that areas of hyperperfusion were associated with future development of dementia in PD, a representative Lewy body diseases^30^, the differential roles of hypo- and hypermetabolism in Lewy body diseases are worth of investigating and require validation in future studies.

This study has several limitations. First, the small sample size might limit the generalizability of the results. Second, this study enrolled patients who were clinically diagnosed with DLB based on recently updated diagnostic criteria^1,2^. Although we enrolled patients with DLB showing presynaptic dopaminergic neuronal degeneration on DAT scans to minimize misdiagnosis, their diagnoses were not pathologically proven. In addition, because 10% of patients with pathologically proven DLB have normal FP-CIT imaging findings^31^, the results of this study should be interpreted cautiously. Third, we did not consider other pathologies such as AD-related pathology. Considering that mixed pathologies are common^32^, future studies should investigate how DLB-hypo and DLB-hyper are affected by amyloid or tau deposition in patients with DLB. Forth, we used whole brain as a reference region for FDG quantification in the present study. Recently, a histogram-based intensity normalized method^33^ has been proposed to detect hyper- and hypo-metabolisms. A histogram-based method revealed quite similar to the areas of hypo-, and hyper- metabolism (Supplementary Fig. 1). However, the method used a group-averaged template from normalized healthy controls, which may require a large number of healthy controls. Therefore, this approach may be not appropriate for our study due to a small subset of heathy control. Also, targeting a specific brain region for normalization is subject to the fact that referenced brain region can be affected in neurodegenerative disease, which could lead to study limitation. Because glucose metabolism of pons, sensori-motor cortex, and cerebellum is known to be affected in patients with DLB^4,29^ and whole brain metabolism was comparable among the groups in the present study (Supplementary Fig. 2), we selected whole brain as a reference region to analyze regional standardized uptake value ratios (SUVRs). Finally, we evaluated longitudinal cognitive decline using MMSE, which showed low sensitivity for detecting cognitive impairment and lacked items assessing executive or visuoperceptual abilities^34^, which are the characteristic neuropsychological deficits in DLB^1^. However, we used detailed longitudinal neuropsychological tests in the cross-sectional analyses, which showed similar results with the longitudinal analyses.

In summary, this study suggests that brain glucose hypo- and hypermetabolism in DLB may be independent of each other and may differentially affect cognitive function in a domain-specific manner. In addition, brain hypometabolism is relevant to longitudinal cognitive outcomes throughout the disease course of DLB, while brain hypermetabolism may be important for cognitive decline in the prodromal status of DLB. Our findings imply that further studies investigating whether brain hypermetabolism in DLB can be a therapeutic target are warranted.

## Methods

### Participants

This study enrolled 13 healthy controls, 15 patients with MCI-LB, and 40 patients with DLB at a university hospital between April 2015 and May 2019. This cohort was used in our previous study^10^. All of the enrolled patients with MCI-LB or DLB fulfilled the research criteria for the clinical diagnosis of probable prodromal DLB^2^ or the 2017 revised diagnostic criteria for DLB, respectively^1^. All patients with DLB showed presynaptic dopaminergic neuronal loss on N-(3-[^18^F]fluoropropyl)-2β-carbomethoxy-3β-(4-iodophenyl) nortropane (FP-CIT) PET scan. The exclusion criteria were as follows: (1) patients with focal brain lesions, severe leukoaraiosis, multiple lacunes in the basal ganglia, or hydrocephalus on brain magnetic resonance imaging (MRI) (*n* = 11); (2) patients with PD^35^ (*n* = 12) or atypical parkinsonism such as multiple system atrophy (*n* = 1), progressive supranuclear palsy (*n* = 2), or corticobasal syndrome (*n* = 2); and (3) patients with other major neurologic (*n* = 1) or psychiatric (*n* = 1) illnesses. All participants in this study underwent neurological examination, detailed neuropsychological testing, 3 T MRI, and FDG PET. All assessments were performed within 3 months. Parkinsonian motor symptoms were assessed during the drug-naïve state at the initial visit using the Unified PD Rating Scale motor subscales. The presence of parkinsonism was determined based on bradykinesia with at least one of rigidity, tremor, or postural instability^36^. Clinical features suggestive of DLB, including cognitive fluctuation, VH, and RBD, were evaluated by patients or caregivers based on semi-structured questionnaires, as described in a previous study^10^. Participants in the control group did not have any subjective symptoms of cognitive impairment or a history of neurological or psychiatric illness. All participants in the control group had normal cognitive function, according to the Korean version of the Mini-Mental State Examination (MMSE > 26) and detailed neuropsychological tests (described later). This study was approved by the institutional review board of Yonsei University College of Medicine (No. 4-2018-0546). Written informed consent was obtained from all participants. In terms of level of data trustworthiness, we believe that data from our earlier study^10^ is trustworthy. Also, enrolled patients with MCI-LB and DLB and healthy controls are anonymized and consented the secondary analyses for other studies. Therefore, we reused these data to investigate our new hypothesis in the present study^37^.

### Acquisition of MRI and FDG-PET

All MRI scans were acquired using a Philips Achieva 3 T scanner (Philips Medical Systems, Best, The Netherlands) with a SENSE head coil (SENSE factor = 2). A high-resolution, T1-weighted MRI volume data set was obtained from all participants with a three-dimensional T1-TFE sequence configured with the following acquisition parameters: axial acquisition with a 224 × 224 matrix; 256 × 256 reconstructed matrix with 182 slices; 220 mm field of view; 0.98 × 0.98 × 1.2 mm^3^ voxels; echo time, 4.6 ms; repetition time, 9.6 ms; flip angle, 8°; and slice gap, 0 mm.

FDG**-**PET acquisition was performed using Discovery 600 (General Electric Healthcare, Milwaukee, MI, USA). All participants were instructed to fast for at least 6 h before the PET/CT scan. A dose of 4.1 MBq of FDG per kilogram of body weight was injected intravenously into the participants. FDG**-**PET images were acquired for 15 min after 40 min after injection. The spiral computed tomography scan was performed with 0.5 s/rotation at 120 kVp, 200 mA, 3.75 mm slice thickness, 10.0 mm collimation and 9.375 mm table feed per rotation. Images were reconstructed using the ordered subset expectation maximization algorithm with four iterations and 32 subsets. A Gaussian filter with 4 mm full-width at half-maximum (FWHM) was applied to the reconstructed PET images, which is a 256 × 256 matrix with 0.98 mm pixel and 0.98 mm slice thickness. All enrolled participants were not taking acetylcholinesterase inhibitors or dopaminergic drugs at the time of MRI and FDG-PET scans.

### Quantification of FDG

Image processing was conducted using Statistical Parametric Mapping 12 (SPM12; Wellcome Trust Center for Neuroimaging, London, UK, https://www.fil.ion.ucl.ac.uk/spm/) software. T1-weighted structural MRI was segmented into gray matter, white matter, and cerebrospinal fluid. FDG PET image was co-registered to the corresponding structural MRI. FDG-PET SUVR image was generated using whole brain uptake. The FDG-PET SUVR image was then warped to the MNI space using a transformation from the corresponding structural MRI. The preprocessed FDG-PET SUVR image was smoothed using a 6 mm FWHM Gaussian kernel.

### Neuropsychological evaluation

All participants underwent a standardized neuropsychological battery called the Seoul Neuropsychological Screening Battery^38^, which contains the following scorable tests: digit span (forward and backward), the Korean version of the Boston Naming Test (K-BNT), Rey-Osterrieth Complex Figure Test (RCFT; copying, immediate recall, 20 min delayed recall, and recognition), the Seoul Verbal Learning Test (SVLT; immediate recall, 20 min delayed recall, and recognition), the semantic (animal and supermarket) and phonemic Controlled Oral Word Association Test (COWAT), and Stroop color reading test. Standardized z-scores were available for all scorable tests based on age- and education-matched norms in 447 healthy controls^38^. The scores in each cognitive domain were classified as abnormal when they were below the 1.5 standard deviation (SD) from the norms^35,39^. MMSE and Clinical Dementia Rating-Sum of Boxes (CDR-SOB) were also measured to assess global cognitive performance^40,41^. Of the 55 patients with MCI-LB or DLB, 48 underwent follow-up MMSE. The average MMSE number and follow-up duration were 3.5 ± 1.2 times and 2.8 ± 1.2 years, respectively. After the diagnosis of MCI-LB or DLB, all patients received acetylcholinesterase inhibitors (donepezil, rivastigmine [patch or capsule], or galantamine) properly during follow-up.

### Statistical analysis

The baseline clinical, neuropsychological, and imaging characteristics of the study participants were analyzed using analysis of variance for continuous variables, whereas chi-square tests or Fisher’s exact tests were used to analyze categorical variables.

To calculate the two metabolic indices reflecting the associated changes in regional cerebral glucose metabolism in DLB, a voxel-wise general linear model was performed using group variables including healthy controls and DLB, and covariates including age, sex, and years of education. The threshold was set at a false discovery rate (FDR)-corrected *P* < 0.05. Significant regions associated with hypometabolism in DLB were identified for DLB-hypo, whereas significant regions related to hypermetabolism in DLB were identified for DLB-hyper. For each patient, the DLB-hypo was extracted from the average SUVR values of the relative hypometabolic regions within an individual gray matter, while the DLB-hyper was extracted from the average SUVR values of hypermetabolic regions within an individual gray matter.

Multivariate linear regression analyses were performed for MMSE scores after adjusting for age at FDG scan, sex, years of education, intracranial volume (ICV), DLB-hypo, and DLB-hyper. Thereafter, multivariate linear regression models were used to investigate the effect of DLB-hypo and DLB-hyper on each item of the neuropsychological test in patients with a DLB spectrum encompassing normal aging, MCI-LB, and DLB. Each cognitive item was included as a dependent variable, and age at FDG scan, sex, years of education, ICV, DLB-hypo, and DLB-hyper were included as independent variables. The selection of variables was performed using the backward elimination method based on the Akaike information criterion. In addition, we tested whether there was an interaction effect between the DLB-hypo and DLB-hyper on cognitive impairment, using the Eq. (1), as aforementioned. Multiple comparisons of the 14 interaction analyses were corrected using the FDR method (*Q* value < 0.05).

Linear mixed models were used to compare the rate of longitudinal changes in total MMSE scores according to baseline DLB-hypo and DLB-hyper in patients with DLB using the Eqs. (2), (3), and (4), as aforementioned. Participants were added as random effects and age, sex, years of education, cognitive status, and ICV as fixed effect terms. The effect of DLB-hypo or DLB-hyper on longitudinal MMSE change over time was tested using an interaction term ([DLB-hypo × time] or [DLB-hyper × time]). To compare the weight of the coefficient per 1 SD between the DLB-hypo and DLB-hyper, we used the individual *z*-transformed values of the two variables in each linear mixed model. A two-way interaction term model was used to investigate the independent effects of each interaction term (*Model 3*). Subgroup analyses according to cognitive status (non-demented and demented subgroups) were also performed using the same statistical models.

Statistical analyses were performed using the R software package (version 4.0; http://www.r-project.org/). Results with *P* < 0.05 and *Q* < 0.05 were considered statistically significant.

### Reporting summary

Further information on research design is available in the Nature Research Reporting Summary linked to this article.

### Supplementary information

Supplementary materials

reporting summary

## Data Availability

The de-identified data that support the findings of this study are available from the corresponding author (P.H.L.) upon request. The data are not publicly available due to privacy or ethical restriction.
