# Supplementary material for: Different effect of hypo- and hypermetabolism on cognition in dementia with Lewy bodies: are they coupled or independent?
Source: NPJ Parkinsons Dis. 2024 Jan 3;10:4. doi: 10.1038/s41531-023-00622-w (PMC10764327; doi:10.1038/s41531-023-00622-w)
Supplement: Supplementary file 1 — Supplementary materials [file 41531_2023_622_MOESM1_ESM.pdf]

Supplementary Figure 1. Topography of DLB-hypo and DLB-hyper using the histogram-based intensity normalization method[1]

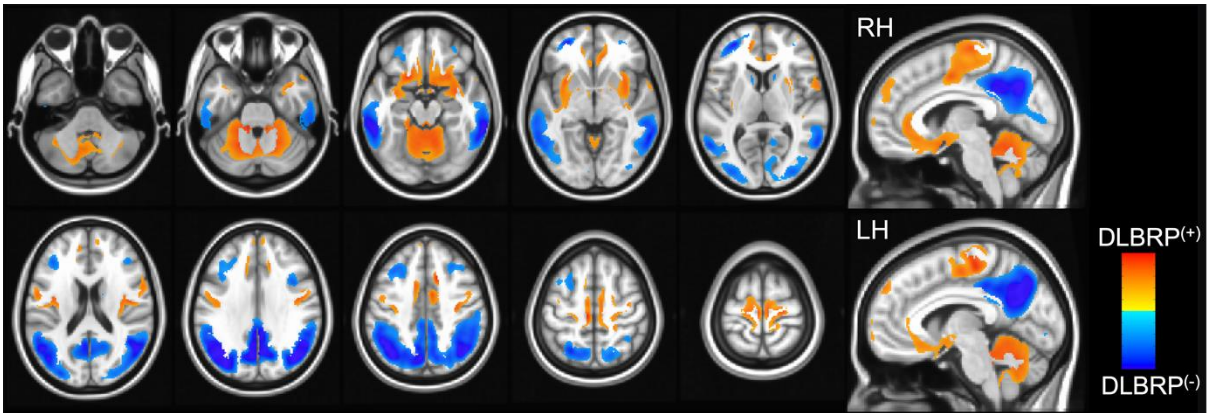

Topography using histogram-based method showed hypo- and hypermetabolic areas in the DLB group compared to normal controls, which were quite similar to Figure 1.

Supplementary Figure 2. Comparison of whole brain SUVRs

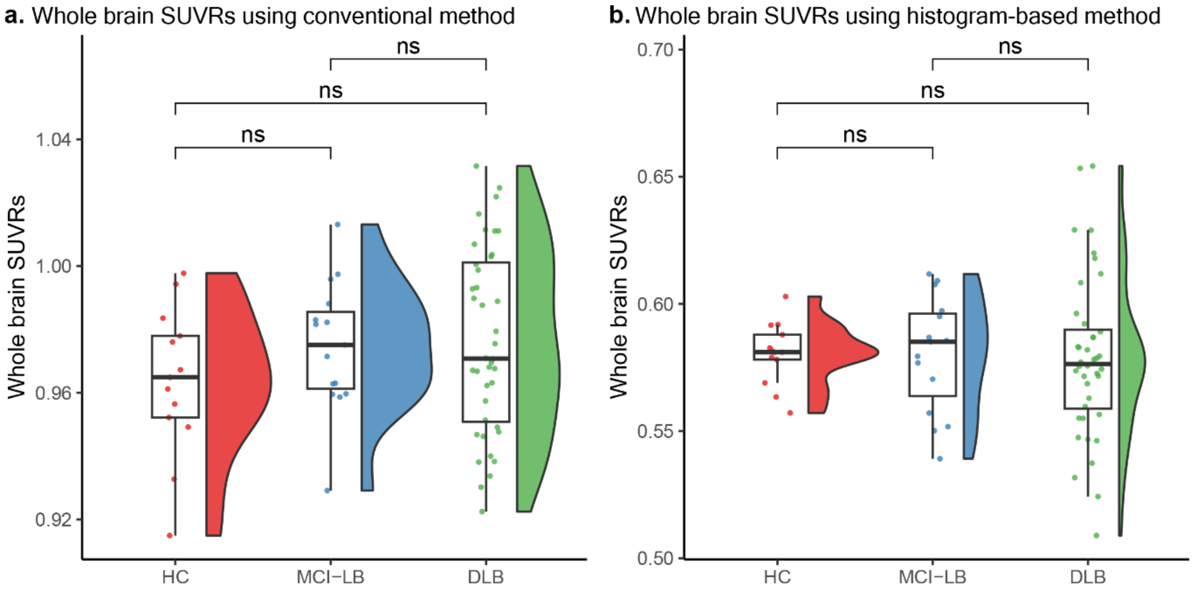

a. conventional method, **b.** histogram-based method. Whole brain SUVRs using conventional method or histogram-based method were comparable among the groups. Centre line denotes median, with bounds of box for quartile and whiskers for the most extreme data point no more than 1.5x the interquartile range from the box.

Supplementary Table 1. Multivariate linear regression analyses for the interaction effect between DLB-hypo and DLB-hyper on each item in neuropsychological test

Cognitive domain	Variables	*β*	*P*	*Q*
Digit span forward	DLB-hypo	1.581	0.557	0.557
	DLB-hyper	0.999	0.663	0.714
	DLB-hypo × DLB-hyper	-1.272	0.636	0.680
Digit span backward	DLB-hypo	3.736	0.155	0.191
	DLB-hyper	2.718	0.225	0.305
	DLB-hypo × DLB-hyper	-3.464	0.187	0.238
K-BNT	DLB-hypo	3.402	0.164	0.191
	DLB-hyper	2.337	0.261	0.305
	DLB-hypo × DLB-hyper	-2.970	0.223	0.260
RCFT copy	DLB-hypo	1.429	0.527	0.557
	DLB-hyper	0.571	0.767	0.767
	DLB-hypo × DLB-hyper	-0.930	0.680	0.680
SVLT immediate recall	DLB-hypo	5.911	0.004	0.013
	DLB-hyper	4.289	0.013	0.044
	DLB-hypo × DLB-hyper	**-5.633**	**0.005**	**0.021**
SVLT delayed recall	DLB-hypo	7.027	0.001	0.005
	DLB-hyper	5.264	0.003	0.020
	DLB-hypo × DLB-hyper	**-6.691**	**0.001**	**0.009**
SVLT recognition	DLB-hypo	3.910	0.086	0.151
	DLB-hyper	2.630	0.175	0.305
	DLB-hypo × DLB-hyper	-3.585	0.115	0.201
RCFT immediate recall	DLB-hypo	7.831	0.000	0.005
	DLB-hyper	6.003	0.001	0.020
	DLB-hypo × DLB-hyper	**-7.333**	**0.001**	**0.009**
RCFT delayed recall	DLB-hypo	6.442	0.003	0.013
	DLB-hyper	4.760	0.009	0.043
	DLB-hypo × DLB-hyper	**-5.918**	**0.006**	**0.021**
RCFT recognition	DLB-hypo	5.526	0.041	0.081
	DLB-hyper	4.277	0.062	0.125
	DLB-hypo × DLB-hyper	**-5.416**	**0.044**	0.088
COWAT animal	DLB-hypo	5.314	0.021	0.057
	DLB-hyper	3.868	0.047	0.114
	DLB-hypo × DLB-hyper	-4.937	0.031	0.082
COWAT supermarket	DLB-hypo	5.460	0.029	0.067
	DLB-hyper	4.183	0.049	0.114
	DLB-hypo × DLB-hyper	-5.246	0.035	0.082
COWAT phonemic	DLB-hypo	3.619	0.127	0.191
	DLB-hyper	2.493	0.216	0.305
	DLB-hypo × DLB-hyper	-3.166	0.180	0.238
Stroop color reading	DLB-hypo	3.330	0.147	0.191
	DLB-hyper	2.207	0.258	0.305
	DLB-hypo × DLB-hyper	-3.122	0.173	0.238

Data are the results of multivariate linear regression models after adjusting for age, sex, education, and intracranial volume.

[a] Q value is the FDR-corrected P value to correct multiple comparisons for 14 cognitive items regression analyses.

Abbreviations: *β* = standardized beta coefficient; COWAT = Controlled Oral Word Association Test; DLB-hypo = hypometabolic changes in dementia with Lewy bodies; DLB-hyper = hypermetabolic changes in dementia with Lewy bodies; FDR = false discovery rate; K-BNT = Korean version of the Boston Naming Test; RCFT, Rey-Osterrieth Complex Figure Test; SVLT = Seoul Verbal Learning Test.

Supplementary Table 2. Baseline demographic characteristics of patients included in the longitudinal analysis

	MCI-LB	DLB	*P* value
Number	15	33	
Demographics			
Age, year	75.19 ± 6.34	76.21 ± 7.77	0.660
Sex, female (%)	3 (20.0)	21 (63.6)	**0.013**
Education, year	11.73 ± 4.18	9.29 ± 5.51	0.134
LBD features			
Cognitive fluctuation	9 (60.0)	18 (54.5)	0.969
Visual hallucination	6 (40.0)	18 (54.5)	0.668
PArkinsonism	10 (66.7%)	23 (69.7%)	>0.999
UPDRS motor score	17.47 ± 5.84	21.77 ± 11.51	0.175
RBD	10 (66.7)	21 (63.6)	>0.999
Vascular risk factors			
Hypertension	6 (40.0)	15 (45.5)	0.846
Diabetes mellitus	3 (20.0)	8 (24.2)	>0.999
Dyslipidemia	5 (33.3)	10 (30.3)	>0.999
Neuropsychological tests			
Item			
Digit span Forward	-0.03 ± 0.76	-0.51 ± 1.02	0.110
Digit span Backward	-0.33 ± 0.88	-1.08 ± 1.26	**0.043**
K-BNT	-0.47 ± 1.01	-1.99 ± 2.08	**0.001**
RCFT copy	-0.84 ± 1.58	-3.16 ± 3.60	**0.003**
SVLT immediate recall	-1.23 ± 0.88	-1.46 ± 1.01	0.458
SVLT delayed recall	-1.41 ± 0.90	-1.94 ± 0.76	0.038
SVLT recognition	-1.52 ± 1.31	-2.00 ± 1.56	0.307
RCFT immediate recall	-1.12 ± 0.78	-1.55 ± 0.60	**0.044**
RCFT delayed recall	-1.19 ± 0.85	-1.75 ± 0.71	**0.021**
RCFT recognition	-0.78 -± 1.32	-1.63 -± 1.15	**0.028**
COWAT animal	-0.99 ± 1.09	-1.65 ± 0.84	**0.028**
COWAT supermarket	-1.14 ± 0.54	-1.41 ± 0.88	0.199
COWAT phonemic	-1.05 ± 0.98	-1.53 ± 0.79	0.151
Stroop color reading	-1.51 ± 1.47	-2.53 ± 1.20	**0.018**
K-MMSE score	25.60 ± 2.06	18.67 ± 4.44	**<0.001**
CDR-SOB	1.77 ± 0.84	5.53 ± 2.05	**<0.001**
FDG-PET			
DLB-hypo	1.17 ± 0.07	1.14 ± 0.08	0.336
DLB-hyper	0.96 ± 0.03	0.97 ± 0.04	0.332

Values are expressed as mean ± standard deviation or number (percentage). P-values are the results of independent *t* test, chi-square tests, or Fisher's exact tests, as appropriate.

Abbreviations: CDR-SOB = Clinical Dementia Rating-Sum of Boxes; COWAT = Controlled Oral Word Association Test; DLB = dementia with Lewy bodies; DLB-hypo = hypometabolic changes in dementia with Lewy bodies; DLB-hyper = hypermetabolic changes in dementia with Lewy bodies; FDG = ^{18}F-fluorodeoxyglucose; K-BNT = Korean version of the Boston Naming Test; K-MMSE = Korean version of Mini-Mental State Examination; MCI-LB = prodromal dementia with Lewy bodies; RBD = rapid eye movement sleep behavior disorder; SVLT = Seoul Verbal Learning Test; UPDRS = Unified Parkinson's Disease Rating Scale; RCFT = Rey-Osterrieth Complex Figure Test

Supplementary Table 3. Longitudinal models predicting change in K-MMSE score over time in the MCI-LB and DLB groups

	Model 1[a]		***Model 2***[b]		***Model 3***[c]	
	**Estimates (SE)**	***P* value**	**Estimates (SE)**	***P* value**	**Estimates (SE)**	***P* value**
In MCI-LB group						
Intercept	40.58 (17.4)	0.044	19.28 (13.22)	0.175	30.79 (16.19)	0.094
Age	-0.23 (0.15)	0.163	-0.07 (0.15)	0.656	-0.13 (0.16)	0.445
Sex, female vs. male	0.55 (2.63)	0.839	0.72 (2.44)	0.772	0.65 (2.34)	0.786
Education	0.29 (0.35)	0.434	0.15 (0.25)	0.577	0.32 (0.30)	0.333
Intracranial volume	-0.83 (9.22)	0.930	5.55 (7.37)	0.470	0.22 (8.04)	0.979
DLB-hypo, per 1 SD increase	0.52 (1.93)	0.790			-0.09 (1.78)	0.961
DLB-hyper, per 1 SD increase			-0.27 (1.21)	0.827	-0.23 (1.21)	0.854
Time, y	-0.69 (0.37)	0.066	-1.19 (0.34)	0.001	-0.86 (0.35)	0.019
DLB-hypo × time	**1.71 (0.64)**	**0.010**			**1.50 (0.61)**	**0.017**
DLB-hyper × time			**-1.27 (0.48)**	**0.011**	**-1.12 (0.46)**	**0.020**
In DLB group						
Intercept	29.82 (16.38)	0.080	25.57 (15.46)	0.110	32.31 (15.98)	0.054
Age	0.00 (0.14)	0.998	0.16 (0.10)	0.131	0.03 (0.14)	0.807
Sex, female vs. male	-2.39 (1.85)	0.206	-2.29 (1.81)	0.217	-2.27 (1.79)	0.217
Education	0.15 (0.18)	0.421	0.14 (0.17)	0.423	0.21 (0.18)	0.251
Intracranial volume	-6.58 (7.85)	0.409	-11.2 (7.94)	0.170	-9.9 (8.02)	0.228
DLB-hypo, per 1 SD increase	0.93 (1.27)	0.472			0.77 (1.27)	0.548
DLB-hyper, per 1 SD increase			-1.31 (1.34)	0.334	-1.15 (1.36)	0.406
Time, y	-1.50 (0.23)	0.000	-1.26 (0.25)	0.000	-1.36 (0.25)	0.000
DLB-hypo × time	**1.07 (0.31)**	**0.001**			**0.85 (0.34)**	**0.014**
DLB-hyper × time			**-0.70 (0.28)**	**0.014**	-0.39 (0.29)	0.182

Data are the results of the linear mixed model for the change in K-MMSE score over time.

[a] Model 1 is the result of the linear mixed model after adjusting for age, sex, education, intracranial volume, DLB-hypo, time, and DLB-hypo × time.

[b] Model 2 is the result of the linear mixed model after adjusting for age, sex, education, intracranial volume, DLB-hyper, time, and DLB-hyper × time.

[c] Model 3 is the result of the linear mixed model after adjusting for age, sex, education, intracranial volume, DLB-hypo, DLB-hyper, time, DLB-hypo × time, and DLB-hyper × time.

Abbreviation: MCI-LB = prodromal dementia with Lewy bodies; DLB-hypo = hypometabolic changes in dementia with Lewy bodies; DLB-hyper = hypermetabolic changes in dementia with Lewy bodies;; K-MMSE = the Korean version of the mini-mental status examination; SE = standard error.

Reference

1 López-González, F. J. *et al.* Intensity normalization methods in brain FDG-PET quantification. *NeuroImage* **222**, 117229, doi:https://doi.org/10.1016/j.neuroimage.2020.117229 (2020).
